# Characterization of Netrin-1 and Its Receptors UNC5B and Neogenin-1 in a Rat Rotator Cuff Tear Model: Associations with Inflammatory Mediators and Neurite Extension

**DOI:** 10.3390/cimb47070511

**Published:** 2025-07-02

**Authors:** Kosuke Inoue, Kentaro Uchida, Mitsuyoshi Matsumoto, Ryo Tazawa, Etsuro Ohta, Akito Hattori, Tomonori Kenmoku, Yuka Ito, Yui Uekusa, Gen Inoue, Masashi Takaso

**Affiliations:** 1 Department of Orthopaedic Surgery, Kitasato University School of Medicine, 1-15-1 Minami-ku, Kitasato, Sagamihara 252-0374, Kanagawa, Japan; inoue.kosuke2@st.kitasato-u.ac.jp (K.I.); kwangyu3244@gmail.com (M.M.); ryotaz@med.kitasato-u.ac.jp (R.T.); kenmoku@med.kitasato-u.ac.jp (T.K.); uekusa.yui@st.kitasato-u.ac.jp (Y.U.); ginoue@kitasato-u.ac.jp (G.I.); mtakaso@kitasato-u.ac.jp (M.T.); 2 Division of Blood Transfusion and Transplantation, Kitasato University School of Health Sciences, Minamiuonuma 949-7241, Nigata, Japan; eohta@kitasato-u.ac.jp (E.O.); hattori.akito@kitasato-u.ac.jp (A.H.); 3 Department of Pathology, Kitasato University School of Allied Health Sciences, Sagamihara 252-0373, Kanagawa, Japan; ito.yuka@st.kitasato-u.ac.jp

**Keywords:** rotator cuff tear, netrin-1, inflammatory cytokine, matrix metalloproteinases

## Abstract

Rotator cuff tears are a leading cause of shoulder pain and dysfunction, yet the molecular mechanisms that link tendon injury to inflammation and nociceptive signaling remain poorly understood. Netrin-1, a classical axon guidance cue signaling through dependence receptors UNC5B and Neogenin-1, has been implicated in both neuronal plasticity and inflammatory processes, but its role in tendon pathology has not been explored. A rat supraspinatus tear model was employed to assess, in vivo, the expression of genes encoding netrin-1 (*Ntn1*) and its receptors (*Unc5b* and *Neo1*) at 0, 7, 14, 28, and 56 days post-injury (n = 10 per time point). Primary rat tenocytes isolated from rotator cuff tissue were treated in vitro with recombinant netrin-1, and transcriptional changes in genes encoding TNF-α (*Tnfa*), IL-6 (*Il6*), MMP-1 (*Mmp1*), and MMP-3 (*Mmp3*) were quantified by qRT-PCR. Separately, human iPSC-derived sensory neurons were exposed to netrin-1, and dose- and time-dependent effects on neurite outgrowth were measured at 4 and 14 days in culture. In injured tendons, *Ntn1* mRNA increased significantly at day 14 (*p* = 0.010) and 28 (*p* = 0.042), *Unc5b* at day 7 (*p* = 0.002) and 14 (*p* < 0.001), and *Neo1* at day 14 (*p* < 0.001) versus intact controls. Tenocyte exposure to 500 ng/mL netrin-1 induced transient upregulation of *Tnfa* (3 h, *p* = 0.023; 6 h, *p* = 0.009) and *Il6* (3 h–24 h, all *p* < 0.013), as well as *Mmp3* (3–24 h, *p* < 0.043) and *Mmp1* (6 h–24 h, *p* < 0.024); no induction was observed at 50 ng/mL. In sensory neurons, 50 ng/mL of netrin-1 enhanced neurite extension at day 4 (*p* = 0.006) but not at 500 ng/mL or at day 14 for either dose. Netrin-1 and its receptors are upregulated in a rat rotator cuff tear model, and netrin-1 elicits distinct pro-inflammatory and matrix-remodeling responses in tenocytes while promoting early neurite growth in sensory neurons. These findings suggest netrin-1 as a key modulator of tendon inflammation, matrix turnover, and peripheral nerve plasticity following injury.

## 1. Introduction

Rotator cuff tears rank among the most prevalent shoulder disorders in older adults, with full-thickness tears affecting approximately 25% of individuals over 60 and nearly 50% by age 80 [1]. Although many tears remain clinically silent, symptomatic tears present with pain, nocturnal discomfort, and progressive muscle weakness, which markedly impairs daily function and quality of life [2,3]. Yet despite this clear clinical burden, the mechanisms by which tendon disruption leads to chronic pain and functional decline remain poorly understood.

Following tendon injury, local inflammatory cascades are activated, driving both matrix degradation and nociceptive sensitization [4,5]. Subacromial bursa specimens from patients with chronic rotator cuff tears contain approximately three-fold higher TNF-α and 3.5-fold higher IL-6 levels than those from control subjects, reflecting local inflammatory activation in rotator cuff disease [4,5]. In an unstabilized rat rotator cuff defect model, gait analysis revealed significant reductions in stride length, print area, and contact intensity—indicators of pain-related behavior—that paralleled marked elevations of TNF-α, IL-1β, and IL-6 in the subacromial bursa and glenohumeral synovium at both 21 and 56 days post-tear, implicating these cytokines in the development and persistence of rotator cuff-associated pain [6]. Inflammatory cytokines also trigger the release of neurogenic sensitizers: in rats, *Ngf* mRNA expression levels increase within 24 h of cuff transection and remain elevated through eight weeks, an effect recapitulated by the TNF-α stimulation of tendon-derived cells [7]. Likewise, IL-1β potently upregulates both NGF and COX-2 in human subacromial bursa cell [8]. These findings underscore the importance of early inflammatory mediators as drivers of tendon pain.

Parallel to cytokine activation, matrix metalloproteinases orchestrate tendon extracellular matrix turnover [9]. Matrix metalloproteinases (MMPs) are a large group of proteolytic enzymes responsible for tissue remodeling and extracellular matrix degradation [10]. Increased expression of MMPs 1 and 9 as well as decreased MMP-3 expression are detected in torn RCT tissue [11]. MMP-1 is a protease that breaks down type I collagen fibers [12]. MMP-3 is involved in the degradation of various collagens and the regulation and activation of MMPs [13]. Despite these advances in understanding cytokine-driven inflammation and MMP-mediated matrix degradation, they do not fully account for the concurrent alterations in tendon innervation and extracellular architecture seen in rotator cuff disease. However, cytokines, neurotrophic factors, and matrix-degrading enzymes alone do not fully explain the integrated changes in nerve sprouting and tissue remodeling characteristic of rotator cuff pathology.

Attention has therefore turned to axon guidance cues as potential master regulators. Netrin-1, a secreted guidance molecule originally defined for its role in axon pathfinding, binds dependence receptors—including UNC5B and Neogenin-1—to orchestrate neuronal growth, survival, and synaptic plasticity [14]. In adult disease models, aberrant netrin-1: DCC signaling drives pathological nerve sprouting and pain: for example, in a rat bone cancer pain model, tumor-derived netrin-1 activates DCC to promote nociceptive neuron sprouting, and DCC inhibition attenuates hyperalgesia [15]. Beyond its neural effects, netrin-1 modulates inflammatory networks by attenuating neutrophil chemotaxis and promoting macrophage retention [16,17], and directly influences matrix turnover in stromal cells by upregulating MMP-9 and downregulating tissue inhibitors of metalloproteinase [18]. These multifaceted actions position netrin-1 as a candidate integrator of the inflammatory, degradative, and neuroplastic responses to tendon injury.

In this study, we employ a rat rotator cuff tear model to test the hypothesis that netrin-1 serves as a central cue at the tendon–nerve interface. Specifically, we will (1) quantify temporal changes in netrin-1, UNC5B, and Neogenin-1 expression in injured supraspinatus tendons; (2) determine how netrin-1 modulates tenocyte production of TNF-α, IL-6, MMP-1, and MMP-3 in vitro; and (3) assess its effects on neurite extension in cultured sensory neurons. By integrating these in vivo and in vitro approaches, we aim to elucidate the mechanistic role of netrin-1 signaling in tendon inflammation, matrix remodeling, and nociceptive sensitization following rotator cuff tears.

## 2. Materials and Methods

### 2.1. Animals

Eight-week-old male Wistar rats (Charles River Laboratories Japan, Yokohama, Japan) were housed in a semi-barrier system under a controlled environment (temperature: 23 ± 2 °C; humidity: 55 ± 10%; 12 h light/dark cycle) with ad libitum access to a commercial pelleted diet (CRF-1; Oriental Yeast Industry, Tokyo, Japan) and water. All procedures conformed to the guidelines of the Science Council of Japan for the care and use of laboratory animals, and were approved by the Kitasato University Animal Ethics Committee (Approval No. 2024-116).

### 2.2. Rat Rotator Cuff Tear Model

The rotator cuff tear model was established as previously described [7]. Rats were anesthetized via intraperitoneal injection of a 1:1:3 mixture of Vetorphale (Meiji Seika, Tokyo, Japan), midazolam (Sand Co., Yamagata, Japan), and Domitor (Nippon Zenyaku, Fukushima, Japan) at a total volume of 0.05 mL per 100 g body weight. Positioned in the lateral decubitus posture, a 1–1.5 cm incision was made over the right shoulder and the deltoid split longitudinally to expose the rotator cuff. The supraspinatus and infraspinatus tendons were sharply transected at their insertions on the greater tuberosity. The deltoid and skin were closed with 5-0 nylon suture (Natsume, Tokyo, Japan). Animals were returned to their cages postoperatively with unrestricted movement. Supraspinatus tendon samples were collected at 0 (intact), 7, 14, 28, and 56 days after injury (n = 10 per time point) for molecular analysis.

### 2.3. Quantitative Real-Time PCR

Harvested supraspinatus tendon specimens were homogenized in TRIzol reagent (Invitrogen, Carlsbad, CA, USA) using a Polytron PE1200 homogenizer (Kinematica AG, Luzern, Switzerland), then centrifuged at 15,000 rpm for 5 min to remove debris. The clarified supernatant (1 mL) was mixed with 200 µL chloroform, vortexed for 15 s, and processed through Maxtract High Density tubes (Qiagen, Hilden, Germany) per the manufacturer’s protocol to isolate total RNA. Tenocyte samples were processed in parallel using a silica-membrane column kit (RNeasy Mini Kit; Qiagen). RNA concentration and purity were determined by spectrophotometry (Denovix DS-11, Wilmington, DE, USA), and only samples with A_260_/A_280_ ratios of 1.8–2.0 were advanced. To remove genomic DNA contamination, all RNA samples were treated with the TURBO DNA-free™ Kit (Invitrogen, Carlsbad, CA, USA) according to the manufacturer’s instructions prior to reverse transcription. For reverse transcription, 500 ng tendon RNA or 200 ng tenocyte RNA was mixed with random hexamer primers and dNTPs, heated to 65 °C for 5 min, then assembled with 5× First-Strand Buffer, 0.1 M DTT, RNaseOUT, and SuperScript III reverse transcriptase (Invitrogen). The reaction was carried out at 50 °C for 60 min, followed by enzyme inactivation at 70 °C for 5 min.

Quantitative PCR was performed in 25 µL reactions containing 2 µL of cDNA, 200 nM of each gene-specific primer (Table 1), and 12.5 µL SYBR Premix Ex Taq™ (Takara, Kyoto, Japan) on a CFX96 real-time PCR system (Bio-Rad, Hercules, CA, USA). Cycling parameters were 95 °C for 3 min, followed by 40 cycles of 95 °C for 10 s and 60 °C for 30 s. Melt-curve analysis confirmed primer specificity. Primer sequences were designed against NCBI-reported mRNA targets using Primer-BLAST and synthesized by Hokkaido System Science (Sapporo, Japan). Relative transcript levels were calculated by the 2^−ΔΔCt^ method, using *Gapdh* as the internal control.

### 2.4. Isolation and Culture of Rotator Cuff–Derived Tenocytes

Tenocytes were isolated from healthy (non-surgical) eight-week-old male Wistar rats. Additional eight-week-old male Wistar rats were euthanized, and bilateral rotator cuff tissues were harvested, minced, and digested in 0.1% collagenase I (Wako Pure Chemical Industries, Osaka, Japan) at 37 °C for 1 h. The cell suspension was filtered, centrifuged, and plated in α-MEM (Gibco) supplemented with 10% fetal bovine serum (FBS; Gibco) on six-well plates. Tenocytes were maintained at 37 °C in a humidified 5% CO_2_ incubator and used at passage 1. Prior to treatment, cells were serum-starved in α-MEM with 1% FBS for 12 h, then exposed to recombinant netrin-1 (R&D Systems, Minneapolis, MN, USA) at the indicated concentrations for 3, 6, or 24 h. Total RNA extraction and qRT-PCR were performed as described above, with primers listed in Table 1.

### 2.5. Effect of Netrin-1 on Sensory Neurons

Human iPSC-derived sensory neurons (ReproCELL, Yokohama, Japan; Cat. No. RCDN001N) were plated on 10 mm glass coverslips precoated with poly-L-ornithine and fibronectin in 24-well plates [19]. Cultures were maintained at 37 °C in 5% CO_2_, with medium refreshed every 3–4 days. After 24 h, neurons were treated with recombinant netrin-1 at the specified concentrations. Morphological images were captured on day 4 using an Olympus CKX53 inverted microscope (Olympus Corporation, Tokyo, Japan). On day 14, cells were fixed in 4% paraformaldehyde and processed for immunofluorescence: they were incubated with anti-TUBB3 (T8660, Sigma-Aldrich, St. Louis, MO, USA) overnight at 4 °C, followed by Alexa Fluor 647-conjugated secondary antibody (A-21242, Invitrogen) for 1 h at room temperature. Nuclei were counterstained with Hoechst 33342 (Sigma-Aldrich). Images were acquired on a Keyence BZ-X810 microscope (Keyence Corporation, Osaka, Japan) with a 20× objective lens (NA 0.45). Neurite lengths were measured on five random fields per coverslip using the NeuronJ plugin in ImageJ (version 14.3; National Institutes of Health, Bethesda, MD, USA; available at https://imagej.nih.gov/ij/) by tracing all neurite branches from the soma. Two wells per condition and ten neurons per well were analyzed in three independent experiments.

## 3. Results

### 3.1. Expression of Netrin-1 and Its Receptors Following Rotator Cuff Injury

**Figure 1:**
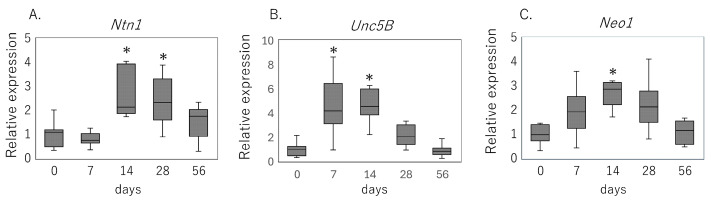
Temporal expression of netrin-1 and its receptors after rotator cuff tear. Relative mRNA levels of (**A**) *Ntn1*, (**B**) *UNC5B*, and (**C**) *Neo1* in rat supraspinatus tendon at 0 (intact), 7, 14, 28, and 56 days following rotator cuff injury, as measured by quantitative RT-PCR. Data are shown as box-and-whisker plots indicating the median, interquartile range, and full range. * *p* < 0.05 versus day 0 (Kruskal–Wallis test with Bonferroni correction).

Supraspinatus tendon analysis demonstrated a coordinated, time-dependent induction of netrin-1 signaling components after rotator cuff tear (Figure 1). *Ntn1* mRNA levels were significantly elevated at day 14 (*p* = 0.010) and remained higher than the intact tendon at day 28 (*p* = 0.042) (Figure 1A). Unc5b transcripts increased as early as day 7 (*p* = 0.002), peaking by day 14 (*p* < 0.001) (Figure 1B), while Neo1 expression was markedly upregulated at day 14 compared with uninjured controls (*p* < 0.001) (Figure 1C).

### 3.2. Inflammatory Cytokine and MMP Expression Following Rotator Cuff Injury

**Figure 2:**
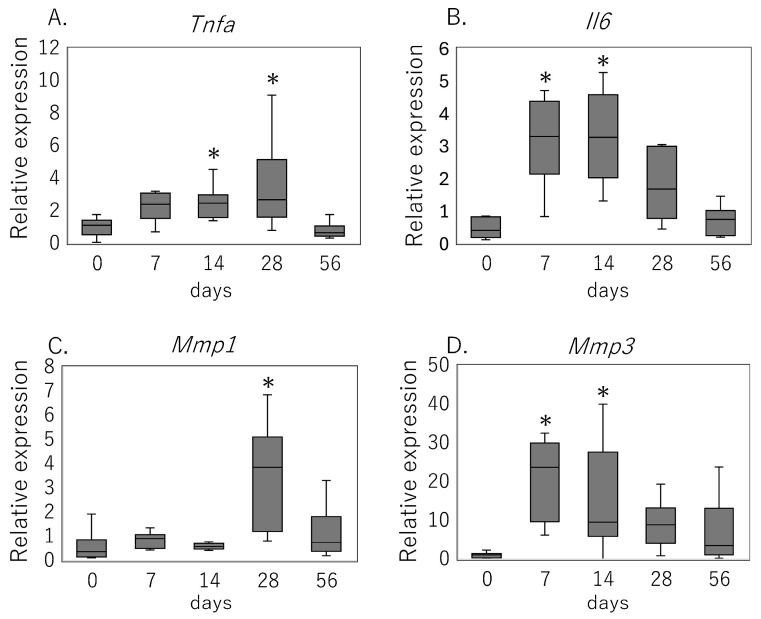
Temporal expression of inflammatory cytokines and MMPs after rotator cuff tear. Relative mRNA levels of (**A**) *Tnfa*, (**B**) *Il6*, (**C**) *Mmp1*, and (**D**) *Mmp3* in rat supraspinatus tendon at 0 (intact), 7, 14, 28, and 56 days following rotator cuff injury, as measured by quantitative RT-PCR. Data are shown as box-and-whisker plots indicating the median, interquartile range, and full range. * *p* < 0.05 versus day 0 (Kruskal–Wallis test with Bonferroni correction).

Key mediators of inflammation and matrix degradation were induced in a distinct temporal pattern during the post-injury period (Figure 2). *Tnfa* expression rose significantly on day 14 (*p* = 0.024) and remained elevated on day 28 (*p* = 0.013) (Figure 2A). *Il6* mRNA showed an early increase by day 7 (*p* < 0.001), which persisted through day 14 (*p* = 0.003) (Figure 2B). Among metalloproteinases, *Mmp3* transcripts were elevated on day 7 (*p* = 0.002) and peaked on day 14 (*p* < 0.001), whereas *Mmp1* upregulation became significant on day 28 (*p* < 0.001) (Figure 2C,D).

### 3.3. Netrin-1–Induced Expression of Cytokines and MMPs in Primary Tenocytes

**Figure 3:**
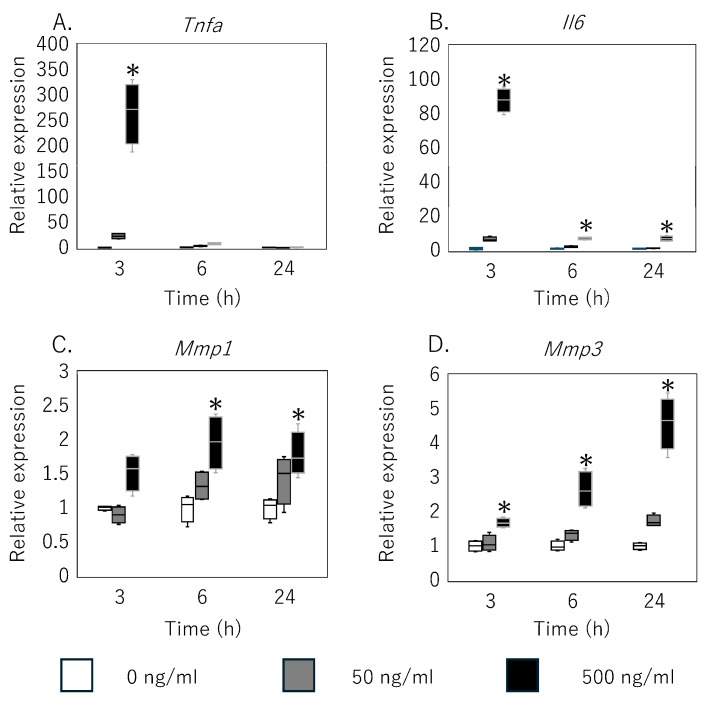
Effect of netrin-1 on cytokine and MMP expression in rat rotator cuff-derived tenocytes. Relative mRNA levels of (**A**) *Tnfa*, (**B**) *Il6*, (**C**) *Mmp1*, and (**D**) *Mmp3* in primary rat rotator cuff tenocytes treated with 0, 50, or 500 ng/mL netrin-1 for 3, 6, and 24 h, as measured by quantitative RT-PCR. Data are shown as box-and-whisker plots indicating the median, interquartile range, and full range. * *p* < 0.05 versus 0 ng/mL at the same time point (Kruskal–Wallis test with Bonferroni correction).

Exposure of primary rat tenocytes to 500 ng/mL netrin-1 elicited a transient upregulation of pro-inflammatory cytokines and matrix-degrading enzymes (Figure 3). *Tnfa* mRNA levels were significantly elevated at 3 h (*p* = 0.023) and 6 h (*p* = 0.009) post-treatment but returned to baseline by 24 h (Figure 3A). *Il6* expression increased at all measured time points—3 h (*p* = 0.005), 6 h (*p* = 0.005), and 24 h (*p* = 0.013) (Figure 3B). Among metalloproteinases, *Mmp1* transcripts were significantly upregulated at 6 h (*p* = 0.013) and remained elevated at 24 h (*p* = 0.024) (Figure 3C). *Mmp3* expression rose as early as 3 h (*p* = 0.043), peaked at 6 h (*p* = 0.007), and persisted through 24 h (*p* = 0.005) (Figure 3D). In contrast, treatment with 50 ng/mL netrin-1 did not induce significant changes in any of these genes at any time point (*p* > 0.05).

### 3.4. Effect of Netrin-1 on Neurite Outgrowth in Human iPSC-Derived Sensory Neurons

**Figure 4:**
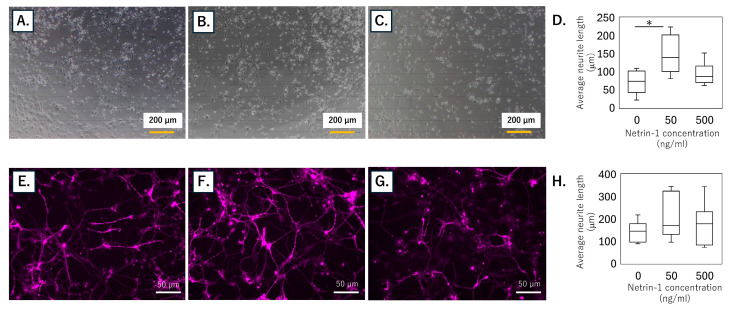
Effect of recombinant netrin-1 on neurite outgrowth in human iPSC-derived sensory neurons. (**A**–**C**) Representative phase-contrast images of neurons at day 4 after treatment with (**A**) vehicle, (**B**) 50 ng/mL netrin-1, or (**C**) 500 ng/mL netrin-1. (**D**) Quantification of total neurite length per neuron at day 4 for vehicle, 50 ng/mL, and 500 ng/mL treatments. (**E**–**G**) Representative fluorescence images of TUBB3-stained neurons at day 14 after treatment with (**E**) vehicle, (**F**) 50 ng/mL netrin-1, or (**G**) 500 ng/mL netrin-1. (**H**) Quantification of total neurite length per neuron at day 14 for vehicle, 50 ng/mL, and 500 ng/mL treatments. Data in (**D**,**H**) are presented as box-and-whisker plots indicating the median, interquartile range, and full range. * *p* < 0.05 versus vehicle (0 ng/mL) (Kruskal–Wallis test with Bonferroni correction).

Neurite extension was quantified at 4 and 14 days in vitro to assess the influence of recombinant netrin-1 (Figure 4). In short-term cultures (day 4), treatment with 50 ng/mL netrin-1 significantly increased total neurite length compared with control (*p* = 0.006), whereas 500 ng/mL exerted no effect (*p* = 1.000) (Figure 4A–D). By day 14, neither dose produced a statistically significant difference in neurite length relative to control (50 ng/mL: *p* = 0.074; 500 ng/mL: *p* = 0.289) (Figure 4E–H).

## 4. Discussion

In this study, we demonstrate for the first time that netrin-1 and its dependence receptors UNC5B and Neogenin-1 are coordinately upregulated in a rat rotator cuff tear model, with peak expression in the subacute phase of tendon injury. Concomitantly, netrin-1 treatment of primary tenocytes elicited a transient increase in pro-inflammatory cytokines (TNF-α, IL-6) and matrix metalloproteinases (MMP-1, MMP-3), whereas cultured sensory neurons exhibited enhanced neurite outgrowth only at early time points and low ligand concentration. These findings collectively implicate netrin-1 as a context-dependent modulator of both tendon cell activation and peripheral nerve plasticity following rotator cuff injury.

The temporal induction of *Ntn1*, *Unc5b*, and *Neo1* transcripts—beginning as early as day 7 for Unc5b and peaking by day 14—parallels not only the onset of tendon degeneration but also the well-documented wave of inflammatory mediator and MMP expression in torn rotator cuff. For example, Yamazaki et al. showed that in their unstabilized rat cuff-defect model, subacromial and synovial levels of TNF-α and IL-6 rise sharply by three weeks and remain elevated through eight weeks, correlating with functional deficits on CatWalk gait analysis inflammatory cytokine [6]. Likewise, clinical specimens from patients with rotator cuff tears exhibit upregulation of TNF-α, IL-1β, and IL-6 in the subacromial bursa, implicating these cytokines in pain generation and chronic inflammation inflammatory cytokine. These temporal overlaps raise the possibility that netrin-1 signaling may be associated with the inflammatory and degradative cascades observed following tendon injury.

A noteworthy observation in this study is that *Unc5b* and *Neo1* were upregulated as early as day 7, preceding the rise in *Ntn1* expression. This temporal mismatch suggests that the early induction of *Unc5b* and *Neo1* is unlikely to be solely a downstream consequence of netrin-1 signaling. Instead, it may reflect the infiltration of receptor-expressing inflammatory cells into the injured tendon during the early post-operative period. For example, Unc5b is known to be constitutively expressed in monocytes, granulocytes, and lymphocytes, and Neo1 expression has been reported in Ly6C^hi^ inflammatory monocytes in sterile peritonitis models [20,21]. Therefore, the increased transcript levels we observed at day 7 may partly result from the accumulation of these immune cell populations in the injured tissue. Additional contributions from resident tenocytes or fibroblasts cannot be excluded, but further studies such as in situ hybridization or cell-type-specific expression analyses will be necessary to determine the cellular source of these receptors. Nevertheless, we acknowledge that our analyses were limited to transcript levels, and that receptor protein abundance or activation status was not assessed. Future studies should aim to quantify receptor expression at the protein level (e.g., by immunostaining or Western blot) and to evaluate receptor activation and downstream signaling in response to netrin-1. Such studies will be essential to determine not only when these receptors are present, but also whether they are functionally engaged in the context of tendon injury.

Matrix remodeling enzymes follow a similarly dynamic pattern. In chronically torn human rotator cuff tissue, Lakemeier et al. observed elevated MMP-1 [11]. Consistent with this, clinical studies report that both MMP-1 levels are significantly higher in patients with rotator cuff tears and that their upregulation is tightly linked to healing failures [22,23]. Moreover, a single investigation has implicated the MMP-3 rs3025058 polymorphism in increased RCT susceptibility, underscoring MMP-3’s importance in tendon integrity [24]. In our rat model, Mmp3 expression rose during the first two weeks post-injury, whereas Mmp1 peaked by day 28—time points that both coincide with significant upregulation of netrin-1 and its receptors. This temporal overlap raises the possibility that netrin-1 signaling contributes to both early MMP-3 induction and later MMP-1 upregulation, potentially by modulating transcriptional pathways governing MMP expression and thereby influencing matrix turnover in injured tendon.

Previous studies have illuminated non-neuronal functions of UNC5B and Neogenin-1 in inflammatory cytokine release [20,25]—roles now extended to resident tenocytes by our data, which show direct TNF-α and IL-6 upregulation in response to netrin-1. Although UNC5B is associated with anti-inflammatory or repulsive signaling, our data suggest that netrin-1-induced cytokine release in tenocytes may be driven primarily through Neogenin-1. In models of renal ischemia–reperfusion injury, netrin-1 engagement of UNC5B shifts T-cell responses toward a Th2–anti-inflammatory phenotype and attenuates tissue damage [20,25]. By contrast, König et al. demonstrated that Neogenin-1 activation on endothelial and immune cells promotes NF-κB-dependent upregulation of pro-inflammatory cytokines and enhances leukocyte recruitment to injured tissue [20,25]. In our tenocyte cultures, the robust induction of TNF-α and IL-6 by netrin-1 therefore likely reflects a dominant netrin-1/Neogenin-1 axis.

Our in vitro neurite outgrowth assays revealed a narrow window in which netrin-1 enhances sensory neuron process extension—specifically, low-dose (50 ng/mL) treatment at day 4—whereas higher concentrations or later time points failed to promote elongation. This biphasic response mirrors the classic dose-dependent actions of axon guidance cues and underscores the critical role of ligand availability in directing neuronal behavior. Notably, high-dose netrin-1 robustly induces TNF-α expression in tenocytes, and elevated TNF-α is well-documented to inhibit neurite outgrowth: Neumann et al. demonstrated that exogenous TNF-α activates RhoA in hippocampal neurons, leading to growth cone collapse and reduced neurite extension via a Rho-dependent mechanism [26], while Cheng et al. showed that glia-derived TNF-α similarly limits cortical neuron process elongation—a suppression reversible by TNF-α neutralization or TNFR1 blockade [27]. Thus, at high netrin-1 doses, the concomitant surge in TNF-α may counterbalance netrin-1’s direct pro-neuritogenic signaling, resulting in an overall inhibitory effect on sensory neurite growth. In this study, we selected 50 ng/mL and 500 ng/mL based on our previous findings using recombinant human netrin-4 [19]. Although this approach enabled the identification of a dose-dependent switch in neurite response, the lack of intermediate concentrations limits the granularity of interpretation. Future studies incorporating a broader concentration gradient will be needed to fully elucidate the dose-response characteristics of netrin-1.

Taken together, these results position netrin-1 as a dual-function signal at the tendon–nerve interface, driving tenocyte-mediated inflammation and matrix remodeling while transiently promoting nociceptive fiber extension. However, it is essential to acknowledge the pleiotropic and sometimes opposing roles of netrin-1 across different biological systems [28]. It should also be noted that the results observed in this study may be highly sensitive to experimental conditions, such as the culture environment, species differences, and receptor expression patterns, all of which can influence the direction and magnitude of netrin-1’s actions.

For instance, administration of secreted netrin family proteins such as netrin-1 and netrin-4 has been reported to prevent bone loss in murine models of skeletal degeneration, suggesting a protective role in chronic bone pathology [29,30]. In contrast, blockade of netrin-1 with a neutralizing antibody attenuated inflammatory bone destruction in models of arthritis and osteolysis [31]. These seemingly contradictory findings likely reflect differences in disease context, receptor expression profiles, ligand availability, and the prevailing immune milieu. Thus, whether netrin-1 promotes or protects against tissue degradation appears to be highly context-dependent.

Indeed, numerous studies have reported anti-inflammatory actions of netrin-1, such as reducing neutrophil infiltration, attenuating pro-inflammatory cytokine production, and promoting the resolution of inflammation in models of peritonitis, colitis, ischemia–reperfusion injury, and sepsis [32,33,34]. These effects are often mediated through UNC5B-dependent pathways and are linked to the enhanced production of anti-inflammatory lipid mediators such as resolvins, or increased presence of alternatively activated macrophages (M2 phenotype). For example, netrin-1 overexpression has been shown to suppress IL-6 and COX-2 expression, reduce Th1/Th2/Th17 cytokines from CD4^+^ T cells, and improve outcomes in models of kidney and cardiac ischemia [35,36].

Conversely, other reports indicate that netrin-1 can have pro-inflammatory or even disease-promoting effects depending on the context. In atherosclerosis, for instance, netrin-1 prevents macrophage egress from plaques, thereby exacerbating lesion development [37]. Similarly, in cancer models, netrin-1 has been implicated in tumor progression and metastasis, potentially by protecting cells from apoptosis and modulating the tumor microenvironment [38].

These apparently conflicting findings underscore the complexity of netrin-1 biology and suggest that its function is highly context- and cell-type-dependent. As Maruyama et al. emphasized, discrepancies in receptor expression, local cytokine milieu [28], and tissue-specific responses can fundamentally alter the direction of netrin-1’s effects. Therefore, our own results—indicating a pro-inflammatory and MMP-inductive effect in tenocytes, but a dose-dependent, transient neurite-promoting effect in sensory neurons—should be interpreted within this broader conceptual framework.

Collectively, these results reinforce the need for caution when considering netrin-1 as a therapeutic target. Its capacity to modulate inflammation and tissue remodeling may be beneficial in some settings, yet detrimental in others. Future studies should aim to define the precise cellular, molecular, and temporal conditions under which netrin-1 exerts protective versus pathogenic effects in the context of tendon injury and pain.

While our findings provide novel insight into netrin-1 signaling in tendon injury, several limitations of the study should be acknowledged. First, our reliance on mRNA endpoints leaves protein expression and receptor activation unverified; future studies should quantify netrin-1 and receptor abundance by immunohistochemistry or Western blot. Second, functional pain behavior and in vivo blockade of netrin-1 signaling (e.g., with receptor antagonists or neutralizing antibodies) will be essential to establish causality between netrin-1 induction and tendon-related pain. Third, although the use of human iPSC-derived sensory neurons provides translational insight, the species difference between these cells and the rat-derived tendon tissues and tenocytes used in the rest of the study introduces a potential incompatibility. While rat and human Netrin-1 share approximately 98% amino acid sequence homology, species-specific differences in cellular context or response cannot be fully excluded. Fourth, while we examined both UNC5B and Neogenin-1 expression levels, this study does not elucidate the distinct biological roles or downstream signaling pathways of each receptor. UNC5B and Neogenin-1 belong to different subclasses of netrin receptors and have been associated with divergent cellular outcomes. A deeper understanding of their individual contributions will require receptor-selective interventions such as gene silencing, overexpression, or pharmacologic inhibition. Finally, although we documented gene expression changes in response to injury and netrin-1 stimulation, our study does not establish functional causality. Future studies incorporating gain- or loss-of-function approaches will be necessary to clarify whether these molecular changes directly mediate tendon healing, inflammation resolution, or neurite outgrowth. Future work incorporating receptor-specific inhibition or gene silencing strategies will be required to delineate their distinct roles in tendon inflammation and repair.

In summary, we provide novel evidence that netrin-1 signaling components are upregulated in injured tendon and that netrin-1 exerts distinct effects on tenocytes and sensory neurons. This work lays the groundwork for further mechanistic and translational studies to unravel how guidance cues such as netrin-1 shape the cellular crosstalk underlying tendon pain and repair.

## 5. Conclusions

Our study provides the first evidence that netrin-1 and its dependence receptors UNC5B and Neogenin-1 are upregulated in the rotator cuff injury, coinciding with the induction of inflammatory cytokines and matrix metalloproteinases that drive tendon degeneration. In vitro, netrin-1 directly stimulated tenocytes to express TNF-α, IL-6, MMP-1, and MMP-3, while exerting a dose- and time-dependent effect on sensory neuron neurite outgrowth. These results implicate netrin-1 as a key mediator at the interface of tendon inflammation, extracellular matrix remodeling, and peripheral nerve plasticity. By illuminating this novel signaling axis, our findings lay the groundwork for future studies to explore targeted modulation of netrin-1 pathways as a strategy to mitigate pain and improve healing after rotator cuff tears.

## Figures and Tables

**Table 1:** Primer sequences used in this study.

Gene		Sequence	bp
*N* *tn1*	sense	TTCTGAAGGCGGACAAAGCA	86
	antisense	GCGTATACGACTTGTGCCCT	
*Unc5B*	sense	GACTCCAAGAACTGCACCGA	172
	antisense	TCCGGTACACGATCACTCCT	
*Neo1*	sense	GAACCCGAGGAAATGCTAGA	105
	antisense	AGGAAGTCCTGAGGCAACAC	
*Tnfa*	sense	CTCTTCTCATTCCCGCTCGT	104
	antisense	GGGAGCCCATTTGGGAACTT	
*Il6*	sense	CCAGTTGCCTTCTTGGGACT	224
	antisense	TCTGACAGTGCATCATCGCT	
*Mmp1*	sense	CTCACACATTCCCACCAGGC	82
	antisense	TTGTCACTGTTGTCGGTCCA	
*Mmp3*	sense	TTGGCACAAAGGTGGATGCT	103
	antisense	TGGGTCACTTTCCCTGCATT	
*Gapdh*	sense	GACTCCAAGAACTGCACCGA	172
	antisense	TCCGGTACACGATCACTCCT	

## Data Availability

The original contributions presented in this study are included in the article. Further inquiries can be directed to the corresponding author(s).

## Abbreviations

The following abbreviations are used in this manuscript:

IL-6	Interleukin-6
MMP	Matrix metalloproteinases
TNF-α	Tumor necrosis factor--α
